# Embolic suppurative thyroiditis with concurrent carcinoma in pregnancy: lessons in management through a case report

**DOI:** 10.1186/s13044-015-0015-5

**Published:** 2015-02-26

**Authors:** Manish M George, Jay Goswamy, Susannah E Penney

**Affiliations:** Department of Otolaryngology-Head and Neck Surgery, Manchester Royal Infirmary, Oxford Road, Manchester, M13 9WL United Kingdom

**Keywords:** Abscess, Suppurative, Thyroiditis, Embolic, Pregnancy, Carcinoma

## Abstract

**Background:**

The thyroid undergoes a variety of physiological changes during pregnancy. The relatively low iodine levels seen in pregnancy have been implicated in thyroid growth during this time. Management of thyroid cancer in pregnancy is not immediately apparent. Furthermore, acute suppurative thyroiditis is rare and this is attributed to the glands innate immunity. We thoroughly review the evidence regarding management of thyroid abscess and thyroid malignancy during pregnancy and illustrate it via an extremely rare case of an embolic thyroid abscess highlighting an underlying carcinoma in a pregnant woman.

**Case:**

A 29-year old female was found to have a thyroid mass during an antenatal assessment. Following a wound infection from Caesarian section she developed a rapidly progressive thyroid abscess. Incision and drainage of the abscess, and subsequent histology revealed papillary carcinoma. She subsequently underwent both total thyroidectomy with level 6 dissection and radio-iodine ablation post-natally.

**Conclusion:**

The literature is inconsistent regarding pregnancy as a risk factor for thyroid cancer, but overall it has been suggested as equally or slightly more frequent than in the non-pregnant population. Thyroid mass investigation should be as for the non-pregnant population. In the first trimester any endocrine surgery is associated with miscarriage, whereas these risks are reduced in second trimester. Importantly, there is no survival benefit in undergoing papillary carcinoma surgery in the third trimester versus early post partum and the risks of premature labour may outweigh any benefit gained by operating early. Most importantly, acute suppurative thyroiditis is rare entity and clinicians should have a low threshold for suspicion of underlying malignancy in these patients. This is especially true in the pregnant population who may be especially susceptible whilst undergoing hypertrophic thyroid changes.

## Background

The thyroid undergoes a variety of physiological changes in pregnancy. High circulating levels of human chorionic gonadotrophin crossbind to the thyroid stimulating hormone (TSH) receptor inducing hypertrophy and hyperplasia [1-4]. A raised level of oestrogen promotes higher levels of thyroid binding globulin and this results in both increased bound thyroxine and a reduction in free thyroxine levels [5,6]. Pregnant women have a high overall total body thyroxine level; however even in this population a low free T4 is inversely correlated with TSH [3]. It is therefore biochemically reasonable that the reduction in free T4 may stimulate a compensatory increase in thyroid activity and growth. Low iodine secretion can cause goitre through decreased thyroxine synthesis and compensatory increased TSH. The relatively low iodine levels seen in pregnancy have been implicated in thyroid growth associated with pregnancy, although this has only been studied in low iodine populations [3,7]. The low incidence of acute suppurative thyroiditis is often attributed to the glands innate immunity. The high local concentration of iodine that inhibits bacterial growth, its degree of lymphatic drainage and its encapsulation all contribute to low local infection rates [8,9]. Dysregulation of these processes via hypertrophy or malignancy may change the susceptibility of the thyroid to infection.

We describe a young woman who developed a thyroid mass during her third trimester of pregnancy. A post-caesarean section wound infection was followed by an episode of acute suppurative thyroiditis. At the time of thyroid abscess drainage, a biopsy was taken of the abscess cavity wall. Papillary thyroid carcinoma was diagnosed. This is the first case in the world literature at the time of publication describing a thyroid abscess of embolic origin. This is also the first report of a postnatal suppurative thyroid gland uncovering a papillary thyroid carcinoma in the acute setting.

## Case report

A 29-year-old female at 37 weeks of an uneventful pregnancy attended antenatal clinic. During her assessment a large predominantly left sided thyroid mass was noted. She was reviewed during that attendance by the local otolaryngology team. She had no history of thyroid pathology, had no lifetime radiation exposure and no relevant family history. There was no palpable cervical lymphadenopathy. Her vocal cords were mobile and symmetrical. Spirometry revealed no intrinsic pulmonary pathology.

An emergency Caesarian section was performed at term + 2 days for fetal distress without intra-operative complication. The child was healthy. Six days post partum she developed a tender erythematous lower abdominal wound, was diagnosed with a wound site infection. A Caesarean section wound swab grew mixed skin flora with anaerobes and treatment was initiated with 625 mg co-amoxiclav. During this time she underwent an ultrasound examination of the thyroid and neck, which revealed a 35 × 40 mm solid nodule within the left thyroid lobe. The left lobe was noted to be hyper-vascular with a surrounding fluid density collection. An outpatient Otolaryngology appointment was made.

She returned to the emergency department the following day with rapid onset tenderness, oedema and erythema over the thyroid. She became haemodynamically unstable with a white cell count of 14.9×10^9^/l and a C-reactive protein of 301 mg/L and was admitted under endocrinology. She was treated with parenterally with 4.5 mg piperacillin/tazobactam three times daily and gentamicin as per trust microbiology advice.

The ultrasound of the thyroid mass was repeated revealing severe expansion of the left lobe with a predominant fluid component. Fine needle aspiration was performed and microbiological assessment again revealed no growth. Cytology however, was suspicious of malignancy. The cytology was repeated and confirmed the likely malignancy.

**Figure 1:**
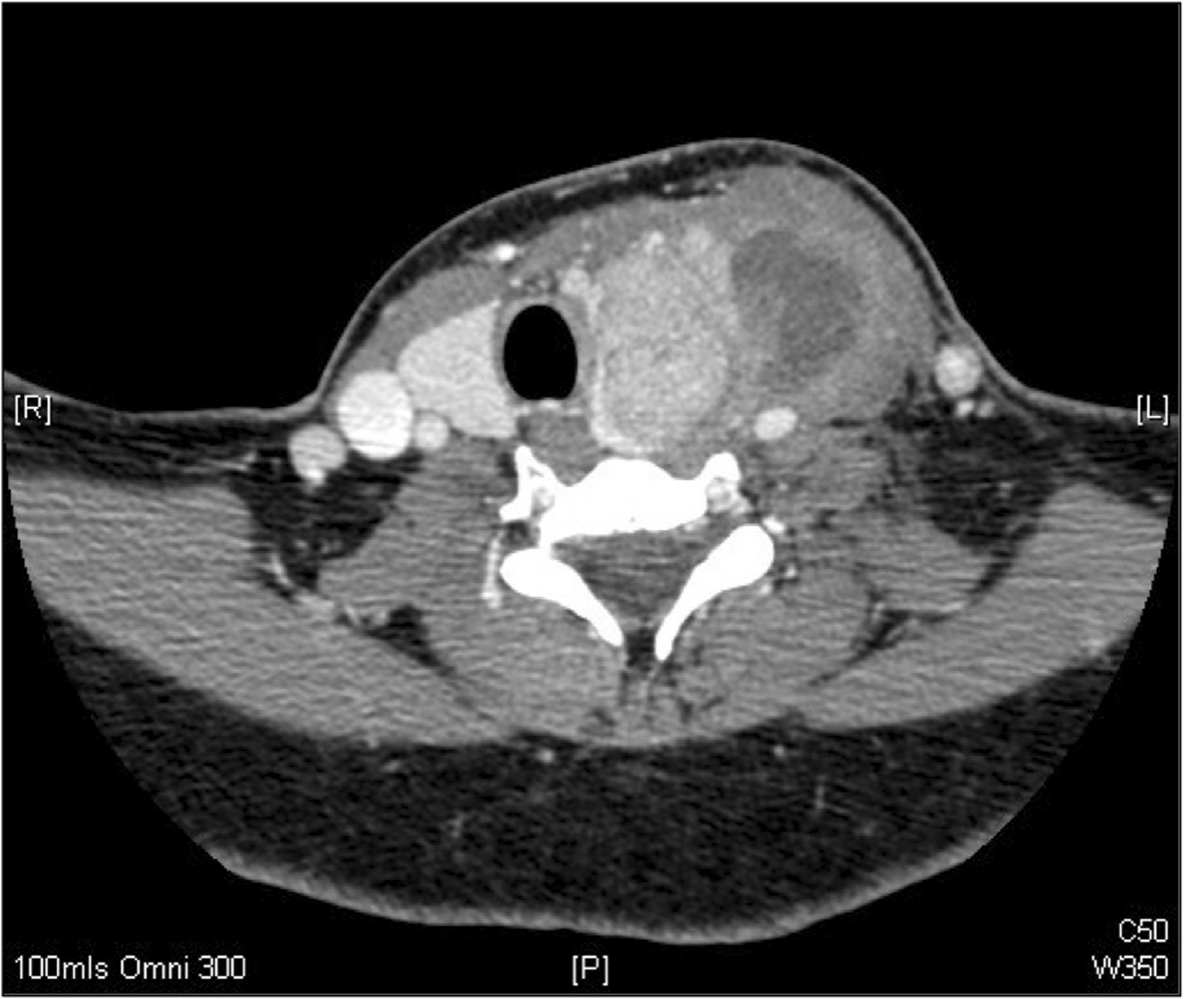
**CT with contrast axial slice through neck.** CT image demonstrating a large fluid collection arising from left thyroid mass, suggestive of an abscess. Noted compression and deviation of adjacent organs.

**Figure 2:**
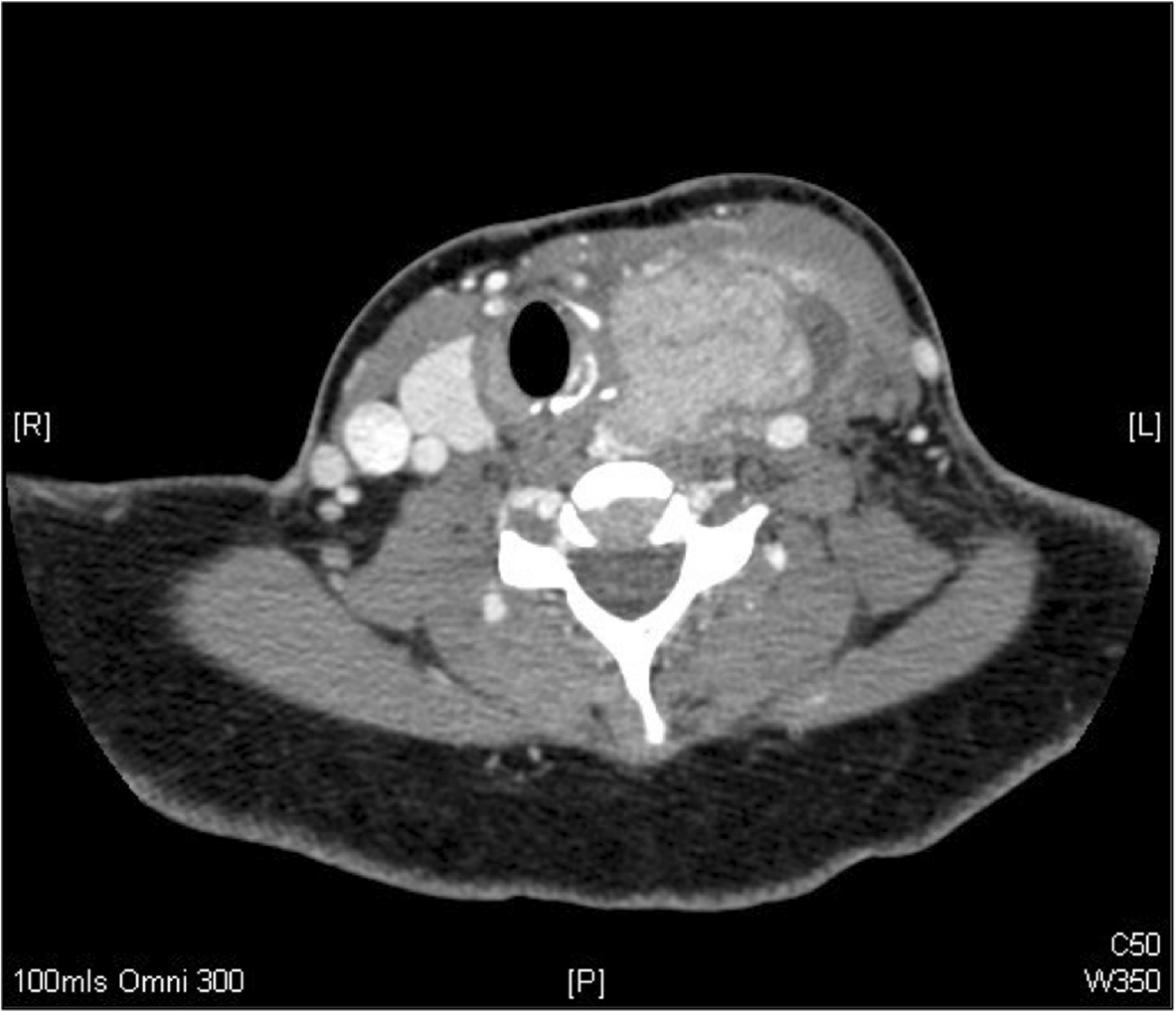
**CT with contrast axial slice through neck.** A more superior CT neck slice demonstrating a resolving abscess and concurrent left thyroid soft tissue mass, now confirmed as a papillary thyroid carcinoma.

Due to a lack of response a computed tomographic scan was performed of her neck (Figure 1). A large enhancing soft tissue mass arising from the left lobe of the thyroid with an area of fluid inferolaterally displacing the surrounding structures was reported. The total size of the lesion including the fluid collection was 60 × 43 × 70 mm. Ipsilateral lymphadenopathy was seen in levels II, III and IV. Clinical staging was cT2N1b (according to the International Union Against Cancer staging). During her acute admission the thyroid abscess was incised and drained and a formal biopsy taken of the intra-thyroidal lesion. Systemic improvement was rapid and she completed a further 48 hours of parenteral co-amoxiclav prior to discharge. Histology revealed a papillary thyroid carcinoma (PTC) and a repeat CT for staging was performed (Figure 2). The left common carotid artery and internal jugular vein were displaced and compressed, but there was no evidence of encasement. The mass extended into the sterno-thyroid and sterno-hyoid muscles and appeared inseparable but not invading. Maximal axial dimensions were 29 × 26 mm, with a maximal coronal dimension of 48 mm. The previously enlarged nodes had resolved.

She subsequently underwent a total thyroidectomy and bilateral level VI clearance. As the previous enlarged nodes had resolved, no other neck dissection was performed. Post-operatively she developed hypocalcaemia and received supplementation, which was reduced over the following 2 months. Histology revealed a 40 mm infiltrative unencapsulated classical PTC with extrathyroid invasion. One of 5 lymph nodes contained metastatic PTC. Her pathological staging was pT3N1a. Post operatively she received radioactive iodine ablation and has made a full recovery.

## Conclusions

There is no consensus in the literature regarding pregnancy as a risk factor for thyroid cancer, but overall it has been suggested as equally or slightly more frequent than in the non-pregnant population [10-16]. There is also no total agreement on pregnancy as a poor prognostic factor for differentiated thyroid cancer, [17,18,14,19,13] the majority of evidence however proposes it does not have a deleterious effect on natural disease progression.

Thyroid mass investigation should be as for the non-pregnant population, that is, with a combination of biochemical, radiological and cytological investigation [20,21]. Some authors do caution that pregnancy induced hyperplasia may make the interpretation of thyroid cytology difficult [22]. Marley and Oertel however, on their cytological analysis of 57 pregnant and post partum women did not find any distinct or differentiating cytological features [10].

The British Thyroid Association guidelines for the management of thyroid cancer suggest two main options for consideration during pregnancy: the first is to defer thyroidectomy and radioiodine until postpartum and the second to defer thyroidectomy until the second trimester with delay of adjuvant radio-iodine ablation until post-partum [23]. It has also been noted that in extreme cases including highly aggressive cancers, possible termination of pregnancy followed by surgery and radio-iodine maybe necessary. In the first trimester any endocrine surgery is associated with miscarriage, whereas these risks are reduced in second trimester [24,25]. Importantly, there is no survival benefit in undergoing papillary carcinoma surgery in the third trimester versus early post partum and the risks of premature labour outweigh any possible marginal benefit gained by operating [21,20,24,18]. It is for this reason that most extensive summary of thyroid disorder in pregnancy and post partum recommends that treatment can be delayed unless the nodule has significantly poor prognosis or is rapidly growing [26]. In cases where cancer is suspected during pregnancy, there is no strong evidence for or against administration of exogenous T4 to suppress TSH levels [27,26].

Thyroid abscesses account for less than 1% of thyroid-related diseases and tend to occur in those with underlying pathology [28,8]. This includes immunosuppression, pre-existing thyroid nodules or underlying malignancy, [8,29-31]. Nishihara et al. report seeding of a thyroid abscess from fine needle aspiration in a patient undergoing regular therapeutic drainage of an adenomatous nodule with cystic degeneration [32]. It is however more commonly due to local direct spread with the most frequent source appearing to be pyriform sinus fistulae [33,30]. Pyriform sinus fistulae related to third and fourth branchial pouch anomalies are a known cause but this tends to present initially in childhood [30,31].

Partial or total thyroidectomy maybe necessary in recurrent, progressive or persistent abscesses, especially in cases with suspicion of underlying malignancy [34,8,35]. Due to concurrent inflammation complete surgical margins may be technically difficult. For this reason it has been recommended that drainage of the abscess acutely with delayed elective partial or total thyroidectomy maybe enable adequate resection margins more frequently [8]. If a pyriform sinus fistula is present a procedure to remove or obliterate the tract is advised [8,36,37].

Acute suppurative thyroiditis has been linked to both oesophageal cancer and laryngeal cancer [35,38]. Concurrent cases of thyroid malignancy are rarely described. In a Saudi report from 2002 Haddad et al. discuss a terminal cardiac event secondary to sepsis from a suppurative infarcted papillary carcinoma found at post mortem [39]. Hong et al. in a Korean publication mention Reidel’s thyroiditis complicated by suppurative thyroiditis and ultimately, micropapillary carcinoma discovered on histology [40]. In our case the papillary carcinoma was not micropapillary, it was suspected before excision and unusually, the abscess was most probably secondary to haematogenous spread via wound infection to a susceptible gland. Intra-operative thyroid pus samples did not grow positive cultures as prolonged course of antibiotics had been administered prior to aspiration. Given the patient’s clear underlying thyroid pathology, once wound infection had been noted, a more cautious approach for monitoring and admission for intravenous antibiotics could have been taken.

### Summary

During pregnancy, evidence and guidelines suggest that optimal management of differentiated cancer is dependent on gestation. As in our case, uncomplicated yet suspicious third trimester masses can be investigated during pregnancy with definitive treatment, namely thyroidectomy, delayed in the vast majority of cases until post-partum.

Thyroid malignancy may predispose to embolic infection, due in part to local dysregulation of the immune system, disruption of the capsule and increased vascularity. The distal lymphatic or haematogenous spread as likely happened in this case is not described in the literature.

Although thyroiditis is a commonly encountered condition, acute suppurative inflammation is less common and may represent underlying malignancy. Drainage with antibiotic therapy maybe sufficient in causing resolution acutely but carcinoma needs to be excluded. Appropriate investigations with expeditious follow up should be employed accordingly.

## Consent

Written informed consent was obtained from the patient for publication of this case report including the accompanying images. A copy of the written consent is available for review by the Editor-in-Chief of this journal.

## References

[1] Haddow JE, McClain MR, Lambert-Messerlian G, Palomaki GE, Canick JA, Cleary-Goldman J (2008). First, Second Trimester Evaluation of Risk for Fetal Aneuploidy Research C. Variability in thyroid-stimulating hormone suppression by human chorionic [corrected] gonadotropin during early pregnancy. J Clin Endocrinol Metab.

[2] Hershman JM (2004). Physiological and pathological aspects of the effect of human chorionic gonadotropin on the thyroid. Best Pract Res Clin Endocrinol Metab.

[3] Elahi S, Hussain Z (2013). A Longitudinal Study of Changes in Thyroid Related Hormones among Pregnant Women Residing in an Iodine Deficient Urban Area. ISRN Endocrinol.

[4] Yoshimura M, Hershman JM (1995). Thyrotropic action of human chorionic gonadotropin. Thyroid.

[5] Chan GW, Mandel SJ (2007). Therapy insight: management of Graves’ disease during pregnancy. Nat Clin Pract Endocrinol Metab.

[6] Lee RH, Spencer CA, Mestman JH, Miller EA, Petrovic I, Braverman LE, Goodwin TM. Free T4 immunoassays are flawed during pregnancy. Am J Obstet Gynecol. 2009;200(3):260 e1-6. doi:10.1016/j.ajog.2008.10.042. 10.1016/j.ajog.2008.10.042 19114271

[7] Kung AW, Chau MT, Lao TT, Tam SC, Low LC (2002). The effect of pregnancy on thyroid nodule formation. J Clin Endocrinol Metab.

[8] Paes JE, Burman KD, Cohen J, Franklyn J, McHenry CR, Shoham S (2010). Acute bacterial suppurative thyroiditis: a clinical review and expert opinion. Thyroid.

[9] Pearce EN, Farwell AP, Braverman LE (2003). Thyroiditis. N Engl J Med.

[10] Marley EF, Oertel YC (1997). Fine-needle aspiration of thyroid lesions in 57 pregnant and postpartum women. Diagn Cytopathol.

[11] Rosen IB, Walfish PG (1986). Pregnancy as a predisposing factor in thyroid neoplasia. Arch Surg.

[12] Rosen IB, Walfish PG, Nikore V (1985). Pregnancy and surgical thyroid disease. Surgery.

[13] Rosvoll RV, Winship T (1965). Thyroid carcinoma and pregnancy. Surg Gynecol Obstet.

[14] Akslen LA, Nilssen S, Kvale G (1992). Reproductive factors and risk of thyroid cancer. A prospective study of 63,090 women from Norway. Br J Cancer.

[15] Kravdal O, Glattre E, Haldorsen T (1991). Positive correlation between parity and incidence of thyroid cancer: new evidence based on complete Norwegian birth cohorts. Int J Cancer.

[16] McTiernan AM, Weiss NS, Daling JR (1984). Incidence of thyroid cancer in women in relation to reproductive and hormonal factors. Am J Epidemiol.

[17] Glinoer D, Soto MF, Bourdoux P, Lejeune B, Delange F, Lemone M (1991). Pregnancy in patients with mild thyroid abnormalities: maternal and neonatal repercussions. J Clin Endocrinol Metab.

[18] Yasmeen S, Cress R, Romano PS, Xing G, Berger-Chen S, Danielsen B (2005). Thyroid cancer in pregnancy. Int J Gynaecol Obstet.

[19] Hod M, Sharony R, Friedman S, Ovadia J (1989). Pregnancy and thyroid carcinoma: a review of incidence, course, and prognosis. Obstet Gynecol Surv.

[20] Moosa M, Mazzaferri EL (1997). Outcome of differentiated thyroid cancer diagnosed in pregnant women. J Clin Endocrinol Metab.

[21] Cooper DS, Doherty GM, Haugen BR, Kloos RT, Lee SL, Mandel SJ (2009). Revised American Thyroid Association management guidelines for patients with thyroid nodules and differentiated thyroid cancer. Thyroid.

[22] Betsill W (1985). Thyroid fine needle aspiration in pregnant women. Diagn Cytopathol.

[23] Perros P, Boelaert K, Colley S, Evans C, Evans RM, Gerrard Ba G (2014). British Thyroid Association: Guidelines for the management of thyroid cancer. Clin Endocrinol (Oxf).

[24] Sam S, Molitch ME (2003). Timing and special concerns regarding endocrine surgery during pregnancy. Endocrinol Metab Clin North Am.

[25] Kuy S, Roman SA, Desai R, Sosa JA. Outcomes following thyroid and parathyroid surgery in pregnant women. Arch Surg. 2009;144(5):399–406; discussion doi:10.1001/archsurg.2009.48. 10.1001/archsurg.2009.48 19451480

[26] De Groot L, Abalovich M, Alexander EK, Amino N, Barbour L, Cobin RH (2012). Management of thyroid dysfunction during pregnancy and postpartum: an Endocrine Society clinical practice guideline. J Clin Endocrinol Metab.

[27] Stagnaro-Green A, Abalovich M, Alexander E, Azizi F, Mestman J, Negro R (2011). Postpartum. Guidelines of the American Thyroid Association for the diagnosis and management of thyroid disease during pregnancy and postpartum. Thyroid.

[28] Al-Dajani N, Wootton SH (2007). Cervical lymphadenitis, suppurative parotitis, thyroiditis, and infected cysts. Infect Dis Clin North Am.

[29] Berger SA, Zonszein J, Villamena P, Mittman N (1983). Infectious diseases of the thyroid gland. Rev Infect Dis.

[30] Miyauchi A, Matsuzuka F, Takai S, Kuma K, Kosaki G (1981). Piriform sinus fistula A route of infection in acute suppurative thyroiditis. Arch Surg.

[31] Yolmo D, Madana J, Kalaiarasi R, Gopalakrishnan S, Kiruba Shankar M, Krishnapriya S (2012). Retrospective case review of pyriform sinus fistulae of third branchial arch origin commonly presenting as acute suppurative thyroiditis in children. J Laryngol Otol.

[32] Nishihara E, Miyauchi A, Matsuzuka F, Sasaki I, Ohye H, Kubota S (2005). Acute suppurative thyroiditis after fine-needle aspiration causing thyrotoxicosis. Thyroid.

[33] Braverman LE, AP F (2013). Sporadic Painless, Painful Subacute and Acute Suppurative Thyroiditis. Werner & Ingbar's the thyroid: a fundamental and clinical text.

[34] Mollar-Puchades MA, Camara-Gomez R, Perez-Guillen V, Benavides-Gabernet M, Gomez-Vela J, Pinon-Selles F (2006). Thyroid hematoma and infectious thyroiditis after a neck injury. Thyroid.

[35] Premawardhana LD, Vora JP, Scanlon MF (1992). Suppurative thyroiditis with oesophageal carcinoma. Postgrad Med J.

[36] Kim KH, Sung MW, Koh TY, Oh SH, Kim IS (2000). Pyriform sinus fistula: management with chemocauterization of the internal opening. Ann Otol Rhinol Laryngol.

[37] Nicoucar K, Giger R, Pope HG, Jaecklin T, Dulguerov P (2009). Management of congenital fourth branchial arch anomalies: a review and analysis of published cases. J Pediatr Surg.

[38] Wilson TD, Pickard BH, Whittam DE (1969). Carcinoma of the larynx masquerading as acute suppurative thyroiditis. Br J Surg.

[39] Haddad FH, Malkawi OM, Omari AA, Izzat AS, Khassrof HM, Faiad LM (2002). Diabetes and infarcted papillary thyroid cancer. Saudi Med J.

[40] Hong JT, Lee JH, Kim SH, Hong SB, Nam M, Kim YS (2013). Case of concurrent Riedel’s thyroiditis, acute suppurative thyroiditis, and micropapillary carcinoma. Korean J Intern Med.

